# Association of Supine Going-to-Sleep Position in Late Pregnancy With Reduced Birth Weight: A Secondary Analysis of an Individual Participant Data Meta-analysis

**DOI:** 10.1001/jamanetworkopen.2019.12614

**Published:** 2019-10-02

**Authors:** Ngaire H. Anderson, Adrienne Gordon, Minglan Li, Robin S. Cronin, John M. D. Thompson, Camille H. Raynes-Greenow, Alexander E. P. Heazell, Tomasina Stacey, Vicki M. Culling, Jessica Wilson, Lisa M. Askie, Edwin A. Mitchell, Lesley M. E. McCowan

**Affiliations:** 1 Department of Obstetrics and Gynaecology, Faculty of Medical and Health Sciences, University of Auckland, Auckland, New Zealand; 2 Department of Paediatrics: Child and Youth Health, Faculty of Medical and Health Sciences, University of Auckland, Auckland, New Zealand; 3 Discipline of Obstetrics, Gynaecology and Neonatology, University of Sydney, Sydney, Australia; 4 Maternal and Fetal Health Research Centre, School of Medical Sciences, Division of Developmental Biology & Medicine, University of Manchester, Manchester, England, United Kingdom; 5 Department of Nursing and Midwifery, School of Human and Health Sciences, University of Huddersfield, Huddersfield, West Yorkshire, England, United Kingdom; 6 Vicki Culling Associates, Wellington, New Zealand; 7 National Health and Medical Research Council Clinical Trials Centre, University of Sydney, Sydney, Australia; 8 Department Obstetrics and Gynaecology, University of Auckland, Auckland, New Zealand

## Abstract

**Importance:**

Supine maternal position in the third trimester is associated with reduced uterine blood flow and increased risk of late stillbirth. As reduced uterine blood flow is also associated with fetal growth restriction, this study explored the association between the position in which pregnant women went to sleep and infant birth weight.

**Objective:**

To examine the association between supine position when going to sleep in women after 28 weeks of pregnancy and lower birth weight and birth weight centiles.

**Design, Setting, and Participants:**

Prespecified subgroup analysis using data from controls in an individual participant data meta-analysis of 4 case-control studies investigating sleep and stillbirth in New Zealand, Australia, and the United Kingdom. Participants were women with ongoing pregnancies at 28 weeks’ gestation or more at interview.

**Main Outcomes and Measures:**

The primary outcome was adjusted mean difference (aMD) in birth weight. Secondary outcomes were birth weight centiles (INTERGROWTH-21st and customized) and adjusted odds ratios (aORs) for birth weight less than 50th and less than 10th centile (small for gestational age) for supine vs nonsupine going-to-sleep position in the last 1 to 4 weeks, adjusted for variables known to be associated with birth size.

**Results:**

Of 1760 women (mean [SD] age, 30.25 [5.46] years), 57 (3.2%) reported they usually went to sleep supine during the previous 1 to 4 weeks. Adjusted mean (SE) birth weight was 3410 (112) g among women who reported supine position and 3554 (98) g among women who reported nonsupine position (aMD, 144 g; 95% CI, −253 to −36 g; *P* = .009), representing an approximate 10-percentile reduction in adjusted mean INTERGROWTH-21st (48.5 vs 58.6; aMD, −10.1; 95% CI, −17.1 to −3.1) and customized (40.7 vs 49.7; aMD, −9.0; 95% CI, −16.6 to −1.4) centiles. There was a nonsignificant increase in birth weight at less than the 50th INTERGROWTH-21st centile (aOR, 1.90; 95% CI, 0.83-4.34) and a 2-fold increase in birth weight at less than the 50th customized centile (aOR, 2.12; 95% CI, 1.20-3.76). Going to sleep supine was associated with a 3-fold increase in small for gestational age birth weight by INTERGROWTH-21st standards (aOR, 3.23; 95% CI, 1.37-7.59) and a nonsignificant increase in small for gestational age birth weight customized standards (aOR, 1.63; 95% CI, 0.77-3.44).

**Conclusions and Relevance:**

This study found that going to sleep in a supine position in late pregnancy was independently associated with reduced birth weight and birth weight centile. This novel association is biologically plausible and likely modifiable. Public health campaigns that encourage women in the third trimester of pregnancy to settle to sleep on their side have potential to optimize birth weight.

## Key Points

**Question:**

As supine maternal position is associated with reduced uterine blood flow, is going to sleep in a supine position in the third trimester associated with reduced birth weight?

**Findings:**

This prespecified subgroup analysis of control participants in an individual participant data meta-analysis found that women at 28 weeks’ gestation or more who usually went to sleep in a supine position gave birth to infants with significantly lower mean birth weights (3410 g vs 3554 g for nonsupine sleep). This finding was independent of variables known to be associated with birth size.

**Meaning:**

A reduction in birth weight associated with third-trimester back sleeping is clinically significant, biologically plausible, and likely modifiable.

## Teaser

This prespecified subgroup analysis of control participants in an individual participant data meta-analysis examines the association between supine vs nonsupine maternal sleeping position in the third trimester of pregnancy and infant birth weight.

## Introduction

Maternal supine position in late pregnancy is associated with significant hemodynamic changes that can result in a reduction in blood flow to the fetus.^1^ Supine maternal going-to-sleep position has recently been found to confer an independent 2.6-fold (adjusted odds ratio [aOR], 2.63; 95% CI, 1.72-4.04) increased risk of late (≥28 weeks’ gestation) stillbirth.^2,3,4,5,6^ Maternal effects of a supine position in late pregnancy include compression of the inferior vena cava^1,7^ and aorta^1,8^ leading to a reduction in maternal cardiac output,^1,7,9^ a reduction in uterine artery blood flow,^10^ and consequently decreased placental perfusion.^11^ Fetal effects associated with supine maternal position include a redistribution of blood circulation with increased flow through the fetal middle cerebral artery^12,13^ and increased fetal quiescence,^14^ suggesting fetal adaptation to mild hypoxic stress. Given that impaired utero-placental flow is associated with fetal growth restriction,^15^ it is plausible that repeated exposure to supine maternal position during sleep in late pregnancy may adversely affect fetal growth.

The initial going-to-sleep position is the sleep position that women maintain for the longest duration throughout the night^16^; therefore, going-to-sleep position is likely to have the greatest impact on blood flow to the developing fetus.

In this prespecified subgroup analysis of the control participants included in an individual participant data (IPD) meta-analysis of going-to-sleep position and risk of late pregnancy stillbirth, we hypothesized that women in the third trimester who reported going to sleep in a supine position during the previous 1 to 4 weeks would have babies with lower birth weight and birth weight centiles compared with women who did not go to sleep in a supine position.

## Methods

We selected women from the control group with ongoing pregnancies from the Collaborative Individual Participant Data Meta-analysis of Sleep and Stillbirth (CRIBSS) study population.^2,3,4,5,6^ This was a 1-stage meta-analysis stratified by study and site. The IPD search strategy, search results, and PRISMA checklist have been published elsewhere,^2,17^ and the CRIBBS study was registered with the PROSPERO register of systematic reviews.^18^ Five international case-control studies that collected data regarding maternal going-to-sleep position and late stillbirth were included in the CRIBBS IPD.^3,4,5,6,19^ Ethical approval was obtained by each individual case-control study.^2^ Each participant in the case-control studies provided written informed consent. Additional approval for the IPD meta-analysis was obtained from the New Zealand Health and Disability Ethics Committee; this approval applied to the study reported here. Reporting of this study followed the Preferred Reporting Items for Systematic Reviews and Meta-analyses (PRISMA) reporting guideline.

Inclusion criteria for the current study were participation in the control group in the CRIBBS IPD study (comprising control participants recruited in 4 case-control studies from 3 high-income countries, New Zealand [2 studies],^5,6^ Australia,^3^ and the United Kingdom,^4^ between June 2006 and March 2016), gestational age at birth collected in weeks and days (to allow accurate calculation of the customized and INTERGROWTH-21st birth weight centiles), gestation at study interview of 28 weeks and 0 days or more, gestation at birth less than or equal to 42 weeks and 6 days, and data available for usual going-to-sleep position up to 4 weeks before the study interview.^20^ A further case-control study that was included in the CRIBBS IPD was excluded from the current analysis as this online survey collected gestational age in completed weeks only.^19^ Individual participants were also excluded if they had missing variables required for calculation of birth weight centiles. There were no missing data for the variables included in the analyses and no imputation was therefore undertaken. In all studies, a detailed face-to-face interview was undertaken with participants during pregnancy. Maternal ethnicity was included in the analyses as ethnicity has been associated with birth weight and fetal growth.^21,22,23^ Ethnicity data were self-reported from the original studies^2,3,4,5^ and harmonized by criteria agreed on by the CRIBSS IPD collaboration: white (includes New Zealand and Australian European, British, Irish, and Romani, and other Europeans), black (includes British Black, African, and Caribbean), South Asian (includes Indian, Pakistani, Bangladeshi, Sri Lankan, Nepali, Bhutanese, Afghan and Maldivian), Southeast and East Asian (includes Chinese, Japanese, Korean, Vietnamese, Malaysian, and Indonesian), Maori, Pacific Islander, and other ethnicity.^17^ Birth weight data were collected after birth from hospital records.

We calculated the centiles for our study population according to INTERGROWTH-21st and customized centiles using published methods.^24,25^ INTERGROWTH-21st centiles are a birth weight standard derived from a low-risk birth cohort and are adjusted for gestation at birth and infant sex.^20^ Customized centiles are based on a fetal growth standard and are adjusted for gestation and infant sex as well as maternal height, weight, parity, and ethnicity.^26^ As adverse perinatal outcomes, including stillbirth, increase with decreasing birth weight and birth weight centiles,^27^ birth weight centiles were also categorized into less than the 10th centile (small for gestational age [SGA]) and less than the 50th centile. Specifically, birth weight less than the 50th centile was included as well as SGA as per our previous analyses of CRIBBS data.^2^ Furthermore, other publications have demonstrated an association with increased risk of stillbirth compared with infants with birth weight greater than the 50th centile.^2,27^ We also included data on large for gestational age (LGA), defined as birth weight greater than the 90th centile for each measure.

For this analysis, maternal going-to-sleep position was the usual position over the previous week,^5^ previous 2 weeks,^3^ or previous month^4,6^ (whichever was longest) and varied by study. Position was recorded as left side, right side, supine, and other (which included variable sides, prone, and propped). For the main analysis, supine was compared with nonsupine. Secondary analysis was performed using all 4 going-to-sleep positions.

Data were available on going-to-sleep position last night and last month for the same participant from 2 of the included studies.^4,6^ Changes in maternal going-to-sleep position over time were therefore investigated in sensitivity analysis using this subset of participants.

### Statistical Analysis

Birth weight and birth weight centiles were compared by maternal going-to-sleep position and adjusted for infant gestational age at birth and at time of interview, infant sex, and maternal age, height, weight, parity, ethnicity, preexisting diabetes, preexisting hypertension, antepartum hemorrhage, gestational hypertensive disorder, gestational diabetes, cigarette smoking, and recreational drug use. To account for possible study differences, multivariable analyses were also adjusted for individual studies as a covariate. For continuous outcomes (birth weight and birth weight centiles), a generalized linear model was used with predicted adjusted means obtained using least-squares means. For binary outcomes (birth weight centile <10th, <50th, and >90th) logistic regression was used, stratified by study, and aORs and 95% confidence intervals were reported. The threshold for statistical significance was set at 2-tailed *P* < .05. Statistical analyses were performed using SAS statistical software version 9.4 (SAS Institute Inc).

## Results

There were 1804 women who were controls in our CRIBBS database, of whom 1760 (97.6%; mean [SD] age, 30.25 [5.46] years) met the eligibility criteria (Figure). Of these women, 57 (3.2%) reported they usually went to sleep supine during the previous 1 to 4 weeks. Demographic characteristics by maternal going-to-sleep position in control participants are reported in Table 1. There were no differences in maternal age, body mass index, ethnicity, and educational status for those who reported going to sleep in a supine position compared with those who went to sleep in a nonsupine position. Women who were not cohabiting were more likely to report going to sleep in a supine position, as were women who had a parity of 1. Gestation at interview was on average 1 week earlier for those who reported supine going-to-sleep position (mean [SD], 35.5 [3.9] vs 36.5 [3.5] weeks’ gestation; difference, −1.01 weeks; 95% CI, −1.94 to −0.08 weeks; *P* = .03), but mean (SD) gestation at birth was 40.0 (1.4) weeks for both groups (Table 1).

**Figure.**
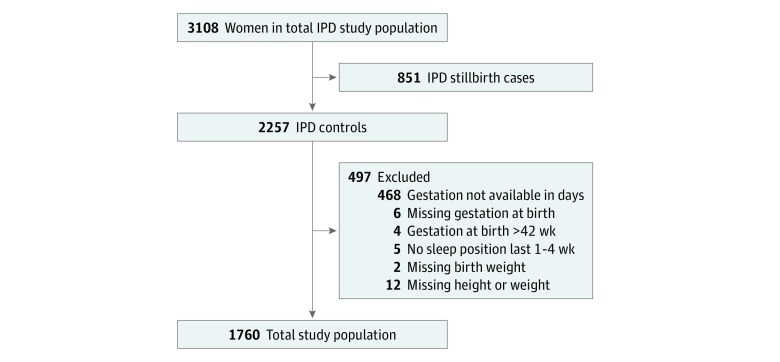
Flowchart of Study Population The eligible population of 3108 excluded women with gestation less than 28 weeks. IPD indicates individual participant data.

**Table 1.** Demographic Characteristics by Maternal Going-to-Sleep Position in Control Participants

Characteristic	Maternal Going-to-Sleep Position		*P* Value
	Nonsupine	Supine	
No. (%)	1703 (96.8)	57 (3.2)	
Individual study			
Auckland	288 (96.3)	11 (3.7)	.38
Sydney	182 (98.4)	3 (1.6)	
New Zealand	541 (95.6)	15 (4.4)	
England	692 (96.1)	28 (3.9)	
Age, mean (SD), y	30.3 (5.5)	29.6 (5.5)	.38
Earliest pregnancy BMI, median (IQR)	24.6 (22.0-29.0)	24.0 (21.0-28.7)	.95
Ethnicity, No. (%)			
White	1074 (97.2)	31 (2.8)	.28
Black	35 (97.2)	1 (2.8)	
South Asian	202 (95.7)	9 (4.3)	
Southeast or East Asian	104 (95.4)	5 (4.6)	
Maori	104 (98.1)	2 (1.9)	
Pacific Islander	143 (94.1)	9 (5.9)	
Other ethnicities	41 (100)	0	
Parity, No. (%)			
0	749 (97.5)	19 (2.5)	.006
1	604 (95.0)	32 (5.0)	
≥2	350 (98.3)	6 (1.7)	
Education, No. (%)			
Primary and/or secondary school	554 (95.5)	26 (4.5)	.12
Trade school	220 (97.3)	6 (2.7)	
Tertiary—university and postgraduate	929 (97.4)	25 (2.6)	
Marital status, No. (%)			
Single	118 (92.2)	10 (7.8)	.002
Married or cohabiting	1585 (97.1)	47 (2.9)	
Preexisting hypertension or diabetes, No. (%)	20 (95.2)	1 (4.8)	.50
Maternal smoking beyond the first trimester, No. (%)	178 (97.8)	4 (2.2)	.52
Recreational drug use during pregnancy, No. (%)	33 (100)	0	.33
Gestation at interview, mean (SD), wk	36.5 (3.5)	35.5 (3.9)	.03
Gestation at birth, mean (SD), wk	40.0 (1.4)	40.0 (1.4)	.87

Abbreviations: BMI, body mass index (calculated as weight in kilograms divided by height in meters squared); IQR, interquartile range.

After adjustment for potential confounding factors, participants who reported they usually went to sleep in a supine position gave birth to infants with an adjusted mean (SE) weight of 3410 (112) g vs 3554 (98) g for participants who reported they usually went to sleep in a nonsupine position, an adjusted mean difference (aMD) of −144 g (95% CI, −253 to −36 g; *P* = .009) (Table 2). Supine going-to-sleep position was also associated with a mean (SE) INTERGROWTH-21st centile of 48.5 (7.1) vs 58.6 (6.2) for nonsupine position (aMD, −10.1; 95% CI, −17.1 to −3.1) and a mean (SE) customized centile of 40.7 (7.6) vs 49.7 (6.7) for nonsupine (aMD, −9.0; 95% CI, −16.6 to −1.4) (Table 2). Supine position was associated with twice the odds of birth weight less than the 50th customized centile (aOR, 2.12; 95% CI, 1.20-3.76). The increase in odds of birth weight less than the 50th INTERGROWTH-21st centile for supine position was not significant (aOR, 1.90; 95% CI, 0.83-4.34) (Table 2). Supine position was associated with a 3-fold increase in odds of SGA by INTERGROWTH-21st centiles (aOR, 3.23; 95% CI, 1.37-7.59), but there was no significant increase in odds of SGA by customized centiles (aOR, 1.63; 95% CI, 0.77-3.44) (Table 2). There was no significant difference in rates of LGA by either birth weight standard between women who went to sleep supine vs nonsupine in the last 1 to 4 weeks of pregnancy.

**Table 2.** Birth Weight, INTERGROWTH-21st Centile, and Customized Centile by Maternal Going-to-Sleep Position

Measure	Maternal Going-to-Sleep Position	
	Nonsupine	Supine
Total study population, No. (%)	1703 (96.8)	57 (3.2)
Birth weight, g^a^		
Mean (SE)	3554 (98)	3410 (112)
aMD (95% CI)		−144 (−253 to −36)
INTERGROWTH-21st centile^b^		
Mean (SE)	58.6 (6.2)	48.5 (7.1)
aMD (95% CI)		−10.1 (−17.1 to −3.1)
INTERGROWTH-21st centile <10th^b^		
No. (%)	76 (4.5)	8 (14.0)
OR	1 [Reference]	3.50 (1.60 to 7.64)
aOR	1 [Reference]	3.23 (1.37 to 7.59)
INTERGROWTH-21st centile <50th^b^		
No. (%)	528 (31.0)	26 (45.6)
OR	1 [Reference]	1.87 (1.10 to 3.18)
aOR	1 [Reference]	1.90 (0.83 to 4.34)
INTERGROWTH-21st centile >90th^b^		
No. (%)	384 (22.6)	10 (17.5)
OR	1 [Reference]	0.73 (0.37 to 1.46)
aOR	1 [Reference]	0.67 (0.32 to 1.41)
Customized centile^c^		
Mean (SE)	49.7 (6.7)	40.7 (7.6)
aMD (95% CI)		−9.0 (−16.6 to −1.4)
Customized centile <10th^c^		
No. (%)	179 (11.0)	9 (15.8)
OR	1 [Reference]	1.60 (0.77 to 3.31)
aOR	1 [Reference]	1.63 (0.77 to 3.44)
Customized centile <50th^c^		
No. (%)	865 (50.8)	39 (68.4)
OR	1 [Reference]	2.10 (1.19 to 3.70)
aOR	1 [Reference]	2.12 (1.20 to 3.76)
Customized centile >90th^c^		
No. (%)	158 (9.3)	3 (5.3)
OR	1 [Reference]	0.54 (0.17 to 1.76)
aOR	1 [Reference]	0.53 (0.16 to 1.70)

Abbreviations: aMD, adjusted mean difference; aOR, adjusted odds ratio; OR, odds ratio.

^a^ Adjusted for study site, gestation, infant sex, and maternal age, height, weight, parity, ethnicity, preexisting diabetes, preexisting hypertension, antepartum hemorrhage, gestational hypertensive disorder, gestational diabetes, cigarette smoking, and recreational drug use.

^b^ Adjusted for study site and maternal age, height, weight, parity, ethnicity, preexisting diabetes, preexisting hypertension, antepartum hemorrhage, gestational hypertensive disorder, gestational diabetes, cigarette smoking, and recreational drug use.

^c^ Adjusted for study site and maternal age, preexisting diabetes, preexisting hypertension, antepartum hemorrhage, gestational hypertensive disorder, gestational diabetes, cigarette smoking, and recreational drug use.

Analysis of all 4 going-to-sleep positions (left side, right side, other, and supine) are shown in Table 3. Birth weight, birth weight centiles, and SGA rates were similar for left, right, and other going-to-sleep positions.

**Table 3.** Birth weight, INTERGROWTH-21st Centile, and Customized Centile by Original Maternal Going-to-Sleep Position

Measure	Original Maternal Going-to-Sleep Position				*P* Value^a^
	Left	Right	Other	Supine	
No. (%)	799 (45.4)	452 (25.7)	452 (25.7)	57 (3.2)	
Birth weight, g^b^					
Adjusted mean (SE)	3552 (99)	3544 (99)	3567 (99)	3410 (112)	
aMD (95% CI)		−8 (−56 to 40)	15 (−35 to 65)	−143 (−253 to −32)	.06
INTERGROWTH-21st centile^c^					
Adjusted mean (SE)	58.2 (6.3)	58.6 (6.3)	58.9 (6.3)	48.4 (7.1)	
aMD (95% CI)		0.5 (−2.6 to 3.5)	0.7 (−2.5 to 3.9)	−9.8 (−16.9 to −2.7)	.04
INTERGROWTH-21st <10th centile					
No. (%)	31 (3.9)	22 (4.9)	23 (5.1)	8 (14.0)	
OR	1 [Reference]	1.27 (0.73 to 2.22)	1.33 (0.77 to 2.31)	4.05 (1.77 to 9.27)	.01
aOR^c^	1 [Reference]	1.05 (0.58 to 1.90)	1.14 (0.62 to 2.09)	3.39 (1.38 to 8.33)	.06
INTERGROWTH-21st <50th centile					
No. (%)	260 (32.5)	136 (30.1)	132 (29.2)	26 (45.6)	
OR	1 [Reference]	0.89 (0.70 to 1.15)	0.86 (0.67 to 1.10)	1.74 (1.01 to 2.99)	.07
aOR^c^	1 [Reference]	0.86 (0.65 to 1.12)	0.80 (0.61 to 1.06)	1.75 (0.97 to 3.16)	.05
INTERGROWTH-21st >90th centile					
No. (%)	181 (22.7)	100 (22.1)	103 (22.8)	10 (17.5)	
OR	1 [Reference]	0.97 (0.74, 1.28)	1.01 (0.77, 1.33)	0.73 (0.36 to 1.47)	.84
aOR^c^	1 [Reference]	0.96 (0.73, 1.27)	1.06 (0.79, 1.42)	0.71 (0.35 to 1.45)	.74
Customized centile^d^					
Adjusted mean (SE)	49.5 (6.7)	49.8 (6.7)	49.6 (6.7)	40.7 (7.6)	
aMD (95% CI)		0.3 (−3.1 to 3.6)	0.1 (−3.4 to 3.5)	−8.9 (−16.6 to −1.1)	.15
Customized centile <10th					
No. (%)	88 (11.0)	50 (11.1)	41 (9.1)	9 (15.8)	
OR	1 [Reference]	1.01 (0.70 to 1.45)	0.81 (0.55 to 1.19)	1.52 (0.72 to 3.19)	.41
aOR^d^	1 [Reference]	0.99 (0.68 to 1.44)	0.84 (0.55 to 1.27)	1.55 (0.72 to 3.35)	.50
Customized centile <50th					
No. (%)	410 (51.3)	225 (49.8)	230 (50.9)	39 (68.4)	
OR	1 [Reference]	0.94 (0.75 to 1.18)	0.98 (0.78 to 1.24)	2.06 (1.16 to 3.65)	.08
aOR^d^	1 [Reference]	0.93 (0.73 to 1.17)	1.00 (0.78 to 1.28)	2.08 (1.16 to 3.72)	.07
Customized centile >90th					
No. (%)	78 (9.8)	40 (8.9)	40 (8.9)	3 (5.3)	
OR	1 [Reference]	0.90 (0.60 to 1.34)	0.90 (0.60 to 1.34)	0.51 (0.16 to 1.68)	.69
aOR^d^	1 [Reference]	0.89 (0.59 to 1.33)	0.90 (0.59 to 1.37)	0.49 (0.15 to 1.63)	.66

Abbreviations: aMD, adjusted mean difference; aOR, adjusted odds ratio; OR, odds ratio.

^a^ *P* values reflect the comparison between the 4 groups.

^b^ Adjusted for study site, gestation at interview and delivery, infant sex, and maternal age, height, weight, parity, ethnicity, preexisting diabetes, preexisting hypertension, antepartum hemorrhage, gestational hypertensive disorder, gestational diabetes, cigarette smoking, and recreational drug use.

^c^ Adjusted for study site and maternal age, height, weight, parity, ethnicity, preexisting diabetes, preexisting hypertension, antepartum hemorrhage, gestational hypertensive disorder, gestational diabetes, cigarette smoking, and recreational drug use.

^d^ Adjusted for study site and maternal age, preexisting diabetes, preexisting hypertension, antepartum hemorrhage, gestational hypertensive disorder, gestational diabetes, cigarette smoking, and recreational drug use.

Within the subset of women who had going-to-sleep position data for both last night and last month (1019 participants), 999 (98.0%) did not change going-to-sleep position between the 2 points. Of the 20 (2.0%) who did change their position, a similar proportion changed from supine to nonsupine (11 women [1.1%]) and from nonsupine to supine (9 women [0.9%]).

## Discussion

In this analysis of women in their third trimester of pregnancy who participated in the control group of CRIBSS, maternal supine going-to-sleep position over the last 1 to 4 weeks was associated with a significant reduction in mean birth weight of 144 g and a 10-percentile reduction in mean INTERGROWTH-21st and customized birth weight centiles. A 3-fold increase in the adjusted odds of SGA by INTERGROWTH-21st centiles was also observed among those who reported they usually went to sleep supine. These reductions in birth weight were independent of variables known to be associated with birth size.

Our finding of similar birth weight and birth weight centiles in all 3 nonsupine going-to-sleep positions (left side, right side, other) is consistent with our previous findings suggesting no difference in stillbirth risk between left side and other nonsupine going-to-sleep positions.^2^

Supine maternal position is associated with a reduction in maternal cardiac output and subsequent fetal blood supply,^1,10^ so it is biologically plausible that supine maternal going-to-sleep position could contribute to reduced birth size. Our finding of an independent mean reduction in birth weight associated with supine going-to-sleep position is clinically relevant. Rates of LGA did not differ between supine and nonsupine groups, but our study may be underpowered to detect a difference. However, rates of LGA in the nonsupine group (22.6% by INTERGROWTH-21st and 9.3% by customized centile) were similar to those reported in general populations.^28^

This is the first study, to our knowledge, to describe the association between supine maternal going-to-sleep position and reduced birth weight in a general obstetric population of women with ongoing pregnancies from a high-income setting. A small observational study^29^ from Ghana reported an increased odds of low–birth weight infants (<2500 g) among maternal supine sleepers but did not report birth weight or birth weight centiles. The study speculated that the association between stillbirth and supine sleep may be mediated by fetal growth restriction.

There is currently no international consensus on the most appropriate way to define normal birth weight; therefore, we elected to investigate 2 commonly used birth weight centiles. For the same infant, customized centiles tend to be lower than INTERGROWTH-21st centiles. This phenomenon has previously been noted^28,30^ and relates to conceptual differences between the birth weight references: INTERGROWTH-21st is a birth weight standard derived from low-risk pregnancies, while customization is a fetal growth standard adjusted for maternal characteristics that influence birth weight. In this study, this is demonstrated by lower mean customized centile and greater numbers of infants with birth weight less than the 10th centile using customized compared with INTERGROWTH-21st centiles. Among nonsupine sleepers, 4.5% of infants were SGA and 22.6% were LGA by the INTERGROWTH-21st standard, compared with 11.0% and 9.3%, respectively, by customized centiles. Despite low numbers of SGA infants in this study, there was a 3-fold increase in odds of SGA by INTERGROWTH-21st centiles in women who reported they usually went to sleep supine (<10th centile: aOR, 3.23; 95% CI, 1.37-7.59) and a nonsignificant increase in SGA by customized centiles. We postulate that the differences in aORs between INTERGROWTH-21st and customized centiles relate to the different location of the distribution of birth weight by each criteria.

Strengths of this study include that it was a prespecified analysis with objective and standardized sleep data and birth weight measurements. In the original case-control studies, sleep position data were collected blinded to the hypothesis, so any bias would be nondifferential. To our knowledge, this is the largest data set assembled with robust data on maternal going-to-sleep position and birth weight.

### Limitations

We acknowledge some limitations with the study. Only a small number of women reported supine sleeping position in late pregnancy, thus limiting power to investigate outcomes in smaller groups such as SGA and LGA. The going-to-sleep position was self-reported; however, it has been demonstrated that there is good correlation between maternal short-term recall of going-to-sleep position and going-to-sleep position recorded by video technology.^16^ Although sleep position changes several times during the night, women spend the longest duration in the position in which they first go to sleep.^31^ Therefore, going-to-sleep position is likely to have the greatest association with fetal blood flow and subsequent associations with birth weight.

The subgroup analysis of women who had going-to-sleep data at 2 points (last night and last month) suggests that the majority of women (97.8%) maintained the same going-to sleep position over the 2 periods, signifying consistency in their exposure. It is also biologically plausible that the association of decreased maternal blood flow on birth size with supine maternal position is cumulative over time. Consequently, increased duration of supine sleeping may lead to greater reduction in birth size. We were not able to investigate this question.

Changing from a supine to a side-lying going-to-sleep position in late pregnancy is a simple intervention that can be easily adopted without known harm^32^ and is applicable to all pregnant women.^2^ Public health campaigns such as those recently launched in both the United Kingdom and New Zealand to encourage women in the third trimester to settle to sleep on their side have potential to optimize birth weight.^33,34^ As the public health message to go to sleep on the side in the third trimester of pregnancy is adopted, further research into the effect of supine maternal going-to-sleep position and birth size is likely to be more difficult to undertake.

## Conclusions

This study found that supine maternal going-to-sleep position is associated with reduced birth size in late pregnancy. Women who reported going to sleep on their back had a clinically relevant and independent reduction in mean birth weight of 144 g, or an adjusted mean reduction of 10% in birth weight centile (customized or INTERGROWTH-21st). Public health campaigns to encourage women to go to sleep lying on their side have potential to increase birth size.
